# The Impact of Mood and Anxiety Disorders on Incident Hypertension at One Year

**DOI:** 10.1155/2014/953094

**Published:** 2014-02-02

**Authors:** Simon L. Bacon, Tavis S. Campbell, André Arsenault, Kim L. Lavoie

**Affiliations:** ^1^Montreal Behavioural Medicine Centre, Research Centre, Hopital du Sacre-Coeur de Montreal, 5400 Boulevard Gouin Ouest, Montréal, QC, Canada H4J 1C5; ^2^Department of Exercise Science, Concordia University, 7141 Sherbrooke Street West, Montreal, QC, Canada H4B 1R6; ^3^Centre de Readaptation Jean-Jacques-Gauthier, Hopital du Sacre-Coeur de Montréal, 5400 Boulevard Gouin Ouest, Montréal, QC, Canada H4J 1C5; ^4^Research Centre, Montréal Heart Institute, 5000 rue Belanger, Montréal, QC, Canada H1T 1C8; ^5^Department of Psychology, University of Calgary, 2500 University Drive NW, Calgary, AL, Canada T2N 1N4; ^6^Department of Psychology, University of Quebec at Montreal (UQAM), C.P. 8888 succ. Centre-ville, Montréal, QC, Canada H3C 3P8

## Abstract

*Background*. Studies assessing the association between psychological factors and hypertension have been equivocal, which may reflect limitations in the assessment of psychological factors. *Purpose*. To assess the relationship between mood and anxiety disorders, measured using a psychiatric interview, and 1-year incident hypertension. *Methods*. 197 nonhypertensive individuals undergoing exercise stress testing at baseline provided follow-up data at 1 year. Baseline assessments included a structure psychiatric interview (PRIME-MD), physician diagnosis of hypertension, and measured blood pressure. At follow-up, hypertension status was assessed via self-reported physician diagnosis. *Results*. Having an anxiety disorder was associated with a 4-fold increase in the risk of developing hypertension (adjusted OR = 4.14, 95% CIs = 1.18–14.56). In contrast, having a mood disorder was not associated with incident hypertension (adjusted OR = 1.21, 95% CIs = 0.24–5.86). *Conclusions*. There are potential mechanisms which could explain our differential mood and anxiety findings. The impact of screening and treatment of anxiety disorders on hypertension needs to be explored.

## 1. Introduction

Hypertension is the leading cause of mortality worldwide [1, 2]. It is a multifactorial disease that can be effectively treated using lifestyle interventions and/or pharmacotherapy. Recent trends indicate that, despite improved detection and treatments, rates of hypertension are not decreasing in Canada [3]. To reverse this trend, we need to improve our understanding of hypertension's aetiology and to determine optimal prevention strategies.

In parallel to hypertension, psychiatric disorders also represent a significant global health burden [4]. Over 25% of the population will develop psychiatric disorders [5]. Mood (depressive) and anxiety (e.g., panic disorder and generalized anxiety disorder) disorders are the most commonly diagnosed disorders, with yearly point prevalences in Canadian adults estimated to be 4.5 and 4.7%, respectively [6]. Furthermore, psychological factors have been shown to be related to worse cardiovascular disease (CVD) outcomes. For example, several meta-analyses have shown that both high depressive symptoms and mood disorders increase the risk of CVD development and progression [7, 8]. Finally, although some have argued that there is considerable covariation between depression and anxiety as risk factors for CVD [9], others have found them to be independent risk factors [10, 11] that increase CVD risk through distinct behavioural and physiological pathways [12].

The fact that hypertension is implicated in the development and progression of CVD and that hypertension seems to be more prevalent in those with higher levels of depression and/or anxiety [13, 14] have led to the hypothesis that hypertension may be an intermediary link between psychopathology and CVD [15]. Mechanistically, this hypothesis also makes sense. Increased levels of both depression and anxiety are associated with health behaviour patterns that increase the risk of developing hypertension (e.g., higher rates of smoking, and less physical activity) [16–19], as well as physiological patterns that are predictive of increased blood pressure (BP) levels (e.g., elevated cardiovascular reactivity during stress and poor cardiovascular recovery following stress) [20–22]. Whilst there is evidence of a link between psychological factors and hypertension development [23–25], not all studies have found this association [26–29]. These inconsistencies may be due to the fact that the majority of studies to date have used questionnaire measures of psychological symptoms rather than interviews that clearly define clinically significant levels of psychological distress (i.e., psychiatric disorders) which can be distinguished from the somatic symptoms related to any underlying physical disease [23, 24, 26–29]. As such, the objective of the current study was to assess the impact of mood and anxiety disorders on the development of hypertension. It was hypothesised that having either a mood or anxiety disorder would be independently associated with the development of hypertension at one year in patients without hypertension at baseline.

## 2. Methods

### 2.1. Participants

The current study is a secondary analysis of the Mechanisms and Outcomes of Myocardial Silent Ischemia (MOMSI) Study [30]. Patients referred for nuclear medicine based exercise stress testing were recruited between May 2005 and December 2006 at the Montreal Heart Institute (MHI). To be eligible, patients had to be ≥18 years of age and English or French speaking. Exclusion criteria included unstable CVD (no cardiac event in the last 4 weeks), a medical condition that conferred greater risk for morbidity than CVD (e.g., cancer), being pregnant or nursing, or suffering from severe psychopathology, current substance abuse, or an apparent cognitive deficit. Of the original 903 MOMSI participants, 357 were considered free of hypertension at baseline. At one year, 197 (55%) of those without baseline hypertension provided questionnaire data.

### 2.2. Procedure

Patients, presenting to the outpatient Nuclear Medicine clinic on the day of their exercise stress test, providing written consent (the study was approved by the Human Research Ethics Board of the MHI), completed a psychiatric interview (see below) followed by a battery of self-report questionnaires assessing sociodemographic and medical history information (including risk factor status). One year after the completion of the baseline assessments, participants were sent a detailed self-report questionnaire which assessed changes in their medical status (including medications). All baseline clinical information was verified via chart review.

### 2.3. Measures

#### 2.3.1. Psychiatric Disorder Assessment: Primary Care Evaluation of Mental Disorders (PRIME-MD)

The PRIME-MD, which is a brief structured psychiatric interview, evaluates some of the most common mental disorders that present in primary care settings. For the purposes of this study, only the mood and anxiety disorder modules were administered, yielding diagnoses for current mood disorders (major depression, minor depression, dysthymia, and bipolar disorder) and anxiety disorders (generalized anxiety disorder, panic disorder, and “other nonspecific” anxiety disorders). The “other anxiety disorders” diagnosis represents probable clinical levels of anxiety without specifying whether the patient meets criteria for specific or social phobia, obsessive-compulsive disorder, or posttraumatic stress disorder, which are the remaining anxiety disorders classified in the Diagnostic and Statistical Manual of Mental Disorders, 4th Edition (DSM-IV) [31]. A computerised version of the PRIME-MD was administered by trained research associates, who were supervised by a licensed clinical psychologist. Using DSM-IV criteria, the PRIME-MD generates diagnoses that have been shown to be of comparable reliability, sensitivity, and specificity as longer structured interviews, such as the Structured Clinical Interview for DSM-IV (e.g., reliability, sensitivity, and specificity for diagnosis of major depressive disorder were *k* = .71, 83%, and 88%, resp.) [31–33]. The PRIME-MD has been used, by us and others, in previous studies assessing psychiatric morbidity in CVD and similar populations [30, 34–37]. Details of the PRIME-MD, including sample questions, can be found at http://www.phqscreeners.com/overview.aspx.

#### 2.3.2. Hypertension Status

Consistent with similar studies [38], at follow-up, those patients who reported a new physician diagnosis of hypertension or had started taking antihypertensive medications were considered to have incident hypertension at one year. Of note, all participants who reported a physician diagnosis of hypertension were also taking an antihypertensive medication at follow-up (i.e., a new prescription).

### 2.4. Data Reduction and Analyses

Missing data was imputed using PROC MI in SAS using the missing at random (MAR) assumptions [39]. Data generated from 5 imputed datasets were averaged to give a single mean estimate and adjusted standard errors according to Harrell's guidelines [40] using the PROC MIANALYZE function. Details of the amount of missing data per variable are included in Table 1. A series of logistic regression models were used to assess the relationship between baseline psychiatric status and incident hypertension. Separate models were generated to assess the main effects of mood disorder and anxiety disorder status, and then a final model including both main effects was computed. Covariates were defined a priori due to their potential influence on the expected relationships under investigation and initially included age, sex, smoking status, and BMI. Additional models were also run adding psychotropic medication. This additional step was included as the pervious literature has suggested that such medications may influence hypertension development [20]. In addition, as part of the review process, we were asked to run the models with a history of CVD included as a further covariate.

## 3. Results

### 3.1. Demographics

Overall, 22% of those who did not have hypertension at baseline had a history of cardiovascular disease (prior myocardial infarction (MI), stroke, heart failure, or revascularisation procedure). With the exception of the presence of an anxiety disorder, there were no baseline differences between those that did and those that did not develop hypertension during the follow-up (as seen in Table 1). However, there was a trend for those who did not develop hypertension to be prescribed psychiatric medications.

### 3.2. Incident Hypertension

As seen in Table 2, there was a significant effect of anxiety disorder on the odds of developing hypertension. Patients with an anxiety disorder at baseline were over 4 times more likely to develop incident hypertension relative to those without an anxiety disorder. This relationship remained statistically significant when all covariates and mood disorder status were adjusted for. Though not reported in Table 2, the inclusion of a history of CVD did not influence the pattern of the analyses and had minimal impact on the final reported point estimates. However, it should be noted that the stability of the point estimates is questionable with the increasing number of covariates included due to the relatively low number of incident cases. Mood disorders were not associated with the development of incident hypertension in any of the models.

## 4. Discussion

The current study found that having an anxiety disorder at baseline was associated with a greater probability of having incident hypertension at one year in patients undergoing single-photon emission computed tomography (SPECT) exercise stress tests. This relationship was independent of important covariates, most notably, having a mood disorder. To our knowledge, this is the first study to prospectively assess the impact of anxiety disorders, measured using a well validated, structured clinical interview, on hypertension incidence. Consistent with our findings, previous studies that have used questionnaires to assess anxiety symptoms have found similar relationships between increased anxiety symptoms and the development of hypertension [28, 41]. Having a mood disorder was not associated with an increased risk of developing hypertension in our population. Our finding of an effect of anxiety but not depression on incident hypertension is consistent with other questionnaire-based studies [42, 43]. However, the depression finding is inconsistent with a recent meta-analysis of prospective studies, including over 22,000 individuals, that found depression to be predictive of hypertension development (relative risk (RR) = 1.42) [13]. However, it should be noted that, with one exception, all of the studies included in this meta-analysis used questionnaires, not a clinical interview, to assess depression, though that one study which did use an interview found major depression to predict incident hypertension [44]. In contrast, Bosworth and colleagues' [25] study of elderly normotensive individuals (which was not included in the meta-analysis above, the reasons for which are not clear) found no relationship between baseline depression status, as measured by the Duke Depression Evaluation Schedule, and hypertensive status over a four-year period. It is also noteworthy that this meta-analysis did not include the largest prospective study of depression and hypertension, where a community sample of over 36,000 individuals (which is over 50% larger than the total sample in the meta-analysis) was followed for 11 and 22 years and found that increased depressive symptoms were associated with lower blood pressure at both follow-ups [29, 45]. From these equivocal reports, it can be seen that there is still much confusion about the potential role of depression in blood pressure changes over time.

Though we did not measure potential mechanisms linking psychiatric disorders to hypertension development, it is possible to speculate on some of the likely explanations for our findings linking anxiety, but not mood, disorders to increased hypertension incidence. It has been suggested that these discrepancies could simply reflect a faster rate of recognition and diagnosis of hypertension in those with anxiety relative to those with depression [46]. This may be due to the fact that patients with anxiety tend to be more likely to present to health care professionals when health concerns or symptoms arise, whereas patients with depression tend to be more avoidant. For example, patients with panic or generalized anxiety disorder (2 prevalent disorders in our study) have a tendency to detect physiological changes at lower thresholds than patients without these disorders and patients with depression [47]. Furthermore, symptoms like decreased motivation and increased fatigue and psychomotor retardation that characterize depression [48] may lead depressed patients to “under-consult” medical services, leading to reduced detection of hypertension.

There are also potential physiological processes that may help to explain our findings. For example, anxiety, but not depression, has been associated with decreased autonomic nervous system function in patients with hypertension [49], including disrupted sympathetic activity in patients with metabolic syndrome and high blood pressure [50]. In addition, having an anxiety disorder or high anxiety symptoms has been associated with worse arterial stiffness [51, 52] and poorer nighttime dipping [53]. Of interest, one study [50] found that the affective symptom component of depression, not the somatic symptom component nor the total depressive symptom score, was associated with altered sympathetic activity in patients with the metabolic syndrome and high blood pressure [50]. The fact that only certain patterns of depressive symptoms were associated with a factor that has been shown to increase the risk of hypertension development may, in part, explain the inconsistent depression-hypertension results seen in the literature.

The results of the current study need to be interpreted taking into account some limitations. First, it is important to note that hypertension diagnosis and medication prescription at one year were self-reported. However, the majority of studies that have assessed the relationship between psychological factors and hypertension have used self-reported outcomes, and there seems to be no systematic difference between those that have used self-reported versus measured blood pressure (i.e., there is still the same level of inconsistency). Whilst we do not believe that the nature of the relationships would change using measured blood pressure, it is likely that the absolute point estimates would be different and the confidence intervals would be smaller than in the current study. However, it is possible that the use of self-reported data could have skewed our results, though the current literature would suggest a low likelihood of this. There is a need for future longitudinal studies that use both interview-derived psychiatric diagnoses and measured blood pressure. Another important limitation is the low number of incident hypertension cases. A one-year incident rate of 8.4% is within the range (4.5–16%) reported in previous researches (e.g., [23, 54]). However, this low absolute number of cases [16] likely accounts for the very large confidence intervals in our data, so caution should be used when interpreting the magnitude of our findings. Though all models were generally consistent with regard to the associations observed, which indicates some degree of robustness, larger studies are needed to provide more accurate point estimates. Finally, it is important to note that our study population was comprised of a group of patients undergoing an exercise stress test, 20% of whom had preexisting CVD. Our findings may therefore not be generalizable to other populations, most notably, patients seen in primary care or the general population. This may also account for the inconsistencies seen in our results in comparison to other studies which have generally included samples more representative of the general population, for example, those from family medicine clinics.

Despite these limitations, this study also has a number of important strengths. It is the first study to use interview-derived measures of both mood and anxiety disorders in a prospective study assessing incident hypertension. One of the key advantages of using an interview compared to self-reported questionnaires is that one is able to disentangle potential confounding of underlying physical disease, for example, CVD, from the psychiatric illness [55]. For example, most depression questionnaires include somatic symptoms, for example, fatigue, loss of energy, and sleep disturbances, which can present as a consequence of CVD rather than depression. If these are endorsed, then the patient may be to generate a score which would be indicative of depression but with no underlying depression related dysfunction. In contrast, using an interview based technique allows for the exploration of the cause of such symptoms and the determination of whether they are based on depression or CVD. Additional strengths include having a well-characterized sample of stable patients without baseline hypertension, the inclusion of a high proportion of women (40%), which improves generalizability, and conducting statistical adjustment for important confounders including mood disorders (anxiety model) and anxiety disorders (mood model), which reduces confounding due to overlapping symptoms.

## 5. Summary

The current study is the first to assess the prospective impact of mood and anxiety disorders on incident hypertension, where it was found that anxiety disorders were associated with an increased risk of developing hypertension. Mood disorders were not associated with incident hypertension.

## Figures and Tables

**Table 1:** Comparison of baseline characteristics in nonhypertensive patients who did and who did not develop hypertension at follow-up data.

	Developed hypertension	Did not develop hypertension	Missing data for follow-up group	*F*	*P*
*N*	16 (8.4%)	174 (91.6%)	7 (4%)*		
Age	58 ± 10	58 ± 10	0 (0%)*	0.02	.889
Sex (women)	4 (25%)	71 (41%)	0 (0%)*	1.53	.218
Any psychiatric disorder	6 (38%)	40 (23%)	0 (0%)*	1.68	.197
Any anxiety disorder	6 (38%)	25 (14%)	0 (0%)*	5.86	.016
Any mood disorder	2 (13%)	25 (14%)	0 (0%)*	0.04	.839
Taking psychiatric medications	0 (0%)	35 (22%)	17 (9%)*	3.87	.051
History of cardiovascular disease	5 (36%)	28 (19%)	27 (14%)	2.37	.126
Taking anti-ischemic medications	0 (0%)	14 (8%)	14 (7%)	1.35	.247
Taking lipid lowering medications	5 (33%)	48 (29%)	14 (7%)	0.15	.699
Taking antiplatelet medications	6 (40%)	56 (33%)	14 (7%)	0.27	.604
Ischemia present on baseline stress test	6 (40%)	53 (31%)	10 (5%)	0.53	.466
BMI	28.1 ± 3.8	26.5 ± 3.8	15 (8%)*	2.45	.120
Smoking status			16 (8%)*	0.54	.462
Never	5 (38%)	55 (34%)			
Previous	8 (62%)	94 (58%)			
Current	0 (0%)	13 (8%)			

*Data used in multiple imputation. BMI: body mass index. A history of cardiovascular disease is defined as a prior myocardial infarction, stroke, heart failure, or revascularisation procedure.

**Table 2:** Association between baseline psychiatric status and incident hypertension at 1 year.

	No covariates		Covariate set 1*		Covariate set 2**	
	OR	(95% CI)	OR	(95% CI)	OR	(95% CI)
Model 1						
Mood disorder	0.84	(0.18−3.92)	0.94	(0.19−4.62)	1.25	(0.24−6.59)
Model 2						
Anxiety disorder	3.50	(1.14−10.77)	5.13	(1.54−17.01)	6.57	(1.82−23.76)
Model 3						
Mood disorder	0.47	(0.09−2.46)	0.57	(0.10−3.17)	0.63	(0.10−3.91)
Anxiety disorder	4.24	(1.29−14.01)	5.65	(1.65−19.35)	7.25	(1.90−27.74)

*Adjusting for age, sex, BMI, and smoking status; **adjusting for age, sex, BMI, smoking status, and psychiatric medication.
